# Safety and Efficacy of Pulsed‐Field Ablation With a Pentaspline Ablation Catheter Versus Cryoablation for Atrial Fibrillation: A Systematic Review and Meta‐Analysis

**DOI:** 10.1002/clc.70459

**Published:** 2026-08-30

**Authors:** Yi Cai, Hao Chen, Miao‐Fu Li, Yi‐Zhou Xu

**Affiliations:** ^1^ The Fourth School of Clinical Medicine Zhejiang Chinese Medical University, Hangzhou First People's Hospital Hangzhou Zhejiang Province China; ^2^ Department of Cardiology, Affiliated Hangzhou First People's Hospital, School of Medicine Westlake University Hangzhou Zhejiang Province China

**Keywords:** atrial fibrillation, cryoablation, meta‐analysis, pulmonary vein isolation, pulsed‐field ablation

## Abstract

**Background:**

Pulsed‐field ablation (PFA) is an emerging technology for atrial fibrillation (AF) ablation. However, there is few studies comparing the clinical advantages and disadvantages of PFA and cryoballoon ablation (CBA). This study aimed to perform a comprehensive meta‐analysis to evaluate the efficacy and safety of PFA using a pentaspline ablation catheter compared with CBA for treating AF.

**Methods:**

This study was registered in the International Prospective Register of Systematic Reviews (ID: CRD420251061599). Studies comparing PFA using a pentaspline ablation catheter with CBA for pulmonary vein isolation (PVI) were systematically searched from database inception to March 2025.

**Results:**

Six trials involving 2024 patients were included. No significant difference was observed in atrial tachyarrhythmia recurrence between PFA and CBA (OR) = 0.71; 95% confidence interval (CI): 0.48 to 1.06). However, in patients with paroxysmal AF, the PFA group exhibited a lower incidence of AT recurrence (OR = 0.62; 95% CI: 0.46 to 0.85). PFA was superior to CBA in terms of ablation time (MD) = −14.12; 95% CI: −16.31 to −11.93) and left atrial dwell time (MD = −9.44; 95% CI: −15.69 to −3.19). Hypersensitive troponin release was higher in the PFA group (MD = 547.29; 95% CI: 53.55 to 1041.03), while CBA was associated with a higher incidence of phrenic nerve palsy (OR = 0.06; 95% CI: 0.01 to 0.30).

**Conclusion:**

PFA demonstrates superior safety and efficacy compared with CBA for PVI in patients with AF. These findings provide additional procedural options for patients and electrophysiologists in clinical practice.

## Graphical abstract

This meta‐analysis compared pentaspline PFA and cryoballoon ablation for AF. PFA shortened procedure time, reduced phrenic nerve injury, and lowered arrhythmia recurrence in paroxysmal AF despite elevated troponin release.

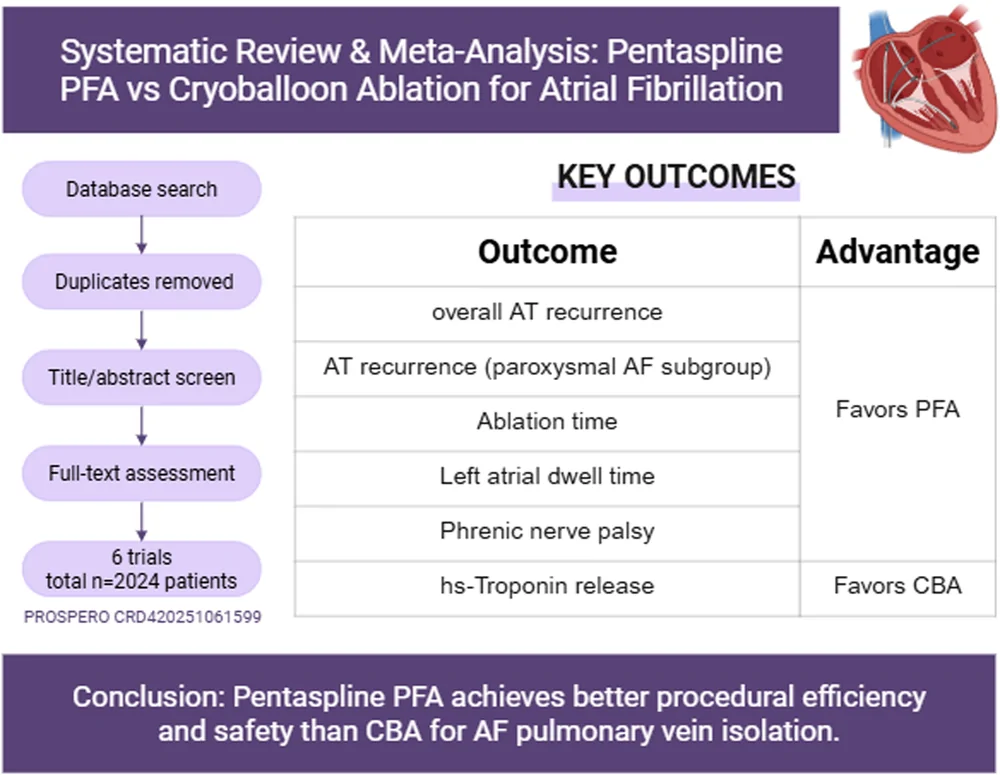

## 1 Introduction

Atrial fibrillation (AF) is the most common arrhythmia, with both incidence and prevalence increasing globally [1]. Approximately 37.574 million cases of AF have been reported worldwide, representing a 33% increase since 1997 [2]. This number is expected to continue to rise over the next 30 years, significantly affecting quality of life and imposing substantial social and healthcare burdens [3]. Current therapeutic strategies for AF primarily rely on catheter ablation (CA), with pulmonary vein isolation (PVI) serving as the cornerstone of this intervention [4]. Evidence indicates that CA reduces AF burden, improves patient quality of life [5], and more effectively maintains sinus rhythm than pharmacological therapy [6].

Common approaches for CA in AF include radiofrequency ablation (RFA), cryoballoon ablation (CBA), and the emerging technique of pulsed‐field ablation (PFA) [7]. RFA remains the most widely used modality; however, its effectiveness depends heavily on operator experience, lacks standardization, and is associated with a relatively higher risk of complications [7]. In contrast, CBA utilizes the thermal properties of rapidly expanding argon gas, which absorbs surrounding heat and induces cellular injury and death at extremely low temperatures [8]. CBA represents an effective alternative to RFA for PVI in AF management, with comparable efficacy and complication rates, while requiring shorter procedural times [8]. PFA induces apoptosis in cardiomyocytes through the application of short‐duration, high‐frequency pulsed electrical fields, leading to irreversible electroporation (IRE) [9]. This technique has attracted increasing attention due to its non‐thermal mechanism, tissue selectivity, and immediate effect, which contribute to improved procedural safety, reduced ablation time, and enhanced durability of PVI [10]. Although multiple meta‐analyses have compared RFA and PFA [11, 12], relatively few studies have directly compared CBA with PFA [13]. Given that both CBA and PFA follow standardized PVI protocols, direct comparison between these approaches allows for a more consistent evaluation of efficacy and safety.

Common PFA catheter types include basket or flower‐shaped, circular, and focal catheters [12]. Among these, the basket or flower‐shaped configuration, exemplified by the pentaspline ablation catheter (FARAWAVE, Boston Scientific), is widely used and enhances the consistency and comparability of clinical outcomes.

While the adoption of PFA and CBA for AF continues to increase, direct comparative studies remain limited and yield inconsistent findings. Therefore, this meta‐analysis aimed to evaluate the efficacy and safety of PFA using a pentaspline ablation catheter compared with CBA in patients with AF, in order to provide evidence to inform clinical practice.

## 2 Materials and Methods

### 2.1 Search Strategy

A systematic search of PubMed, Embase, Scopus, Web of Science, and the Cochrane Library was conducted to identify controlled clinical studies comparing PFA and CBA for initial ablation in patients with AF from database inception to March 2025. The search was restricted to studies published in English. For studies with overlapping patient populations, the publication providing the most comprehensive data was included to avoid duplication. Identical core search terms were used across all databases, including “pulsed‐field ablation,” “cryoballoon ablation,” “thermal ablation,” “atrial fibrillation,” and “catheter ablation.”

### 2.2 Inclusion and Exclusion Criteria

The inclusion criteria were as follows: (1) studies formally published in full‐text English; (2) studies comparing outcomes between patients treated with PFA using a pentaspline ablation catheter and those treated with CBA for PVI; (3) study designs including retrospective studies or randomized controlled trials (RCTs); and (4) studies reporting relevant outcomes.

The exclusion criteria were as follows: (1) observational studies without a control group; (2) duplicate publications; and (3) meta‐analyses, literature reviews, non‐human studies, case reports, editorials, and dissertations.

### 2.3 Data Extraction and Quality Evaluation

Two investigators independently screened the literature according to predefined inclusion and exclusion criteria, extracted relevant data, and cross‐checked the results. Discrepancies were resolved through discussion, and a third investigator was consulted when necessary. Data were systematically extracted using a predefined data extraction form. The following information was collected from each study: participant characteristics, including mean age, sex, body mass index (BMI), hypertension, coronary artery disease, diabetes mellitus, and left ventricular ejection fraction, as well as study characteristics, including author, publication year, study population, sample size, intervention strategy, outcome assessment methods, and follow‐up duration. The Newcastle–Ottawa Scale was used to assess the risk of bias in cohort studies.

### 2.4 Study Endpoints

The primary endpoints included postoperative atrial tachyarrhythmia (AT) recurrence, procedure time, fluoroscopy time, ablation time, left atrial dwell time, and complications, such as phrenic nerve palsy (PNP), cardiac tamponade, and vascular or cerebrovascular events. The secondary endpoints included 12‐month post‐procedural antiarrhythmic drug (AAD) reutilization rates, hypersensitive troponin (hs‐Tn) release, and pulmonary vein (PV) reconnection.

### 2.5 Statistical Analysis

Meta‐analysis was performed using Review Manager (RevMan) version 5.4. Continuous variables were expressed as mean differences (MDs), and dichotomous variables were expressed as odds ratios (ORs). Pooled effect sizes with corresponding 95% confidence intervals (CIs) were calculated.

Heterogeneity among studies was assessed using the *Q* test and quantified using the *I* ^2^ statistic. When *p* > 0.1 or *I* ^2^ ≤ 50%, heterogeneity was considered low, and a fixed‐effects model was applied. When *p* ≤ 0.1 or *I* ^2^ > 50%, significant heterogeneity was assumed, and a random‐effects model was used.

## 3 Results

### 3.1 Search Results and Study Characteristics

**Figure 1:**
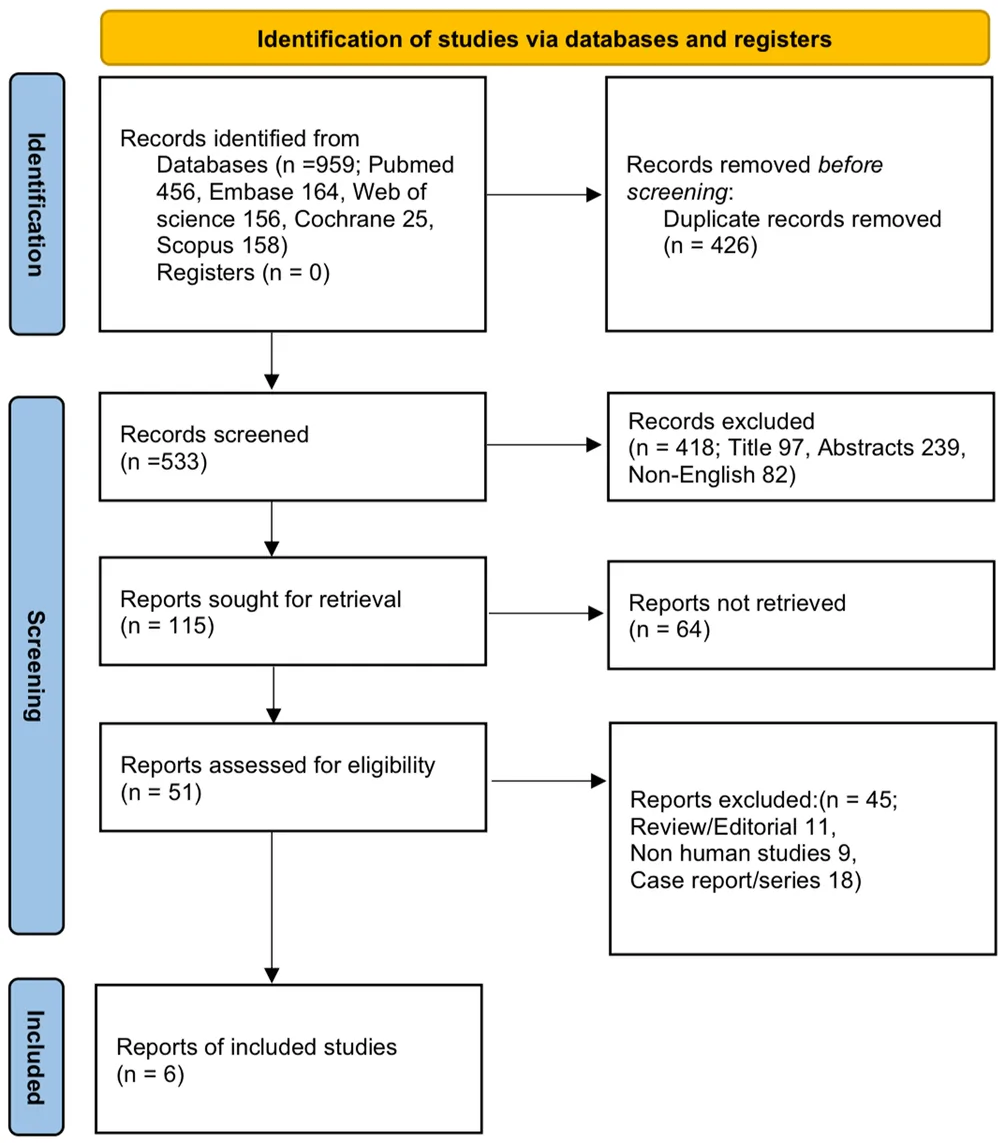
Flow chart of literature search and screening.

The study selection process is illustrated in Figure 1. A total of six trials [14, 15, 16, 17, 18, 19], comprising 2024 patients (996 in the PFA group and 1028 in the CBA group), were included. These studies were published between 2023 and 2025 and consisted of one retrospective cohort study [16] and five RCTs [14, 15, 17, 18, 19].

Baseline characteristics of the included studies are summarized in Table 1. Three studies included patients with paroxysmal AF [14, 15, 16], two focused on persistent AF populations [18, 19], and one included a broader spectrum of AF patients [17]. All participants underwent PVI as their initial ablation procedure.

**Table 1:** Characteristics of the included studies.

Study, year	Study design	Number of cases		Age (years)		Gender (male/female)		BMI (kg/m^2^)		Hypertension (NO.)		Diabetes (NO.)		CAD (NO.)		LVEF (%)		CHA_2_DS_2_‐VASc		Outcomes
		PFA	CBA	PFA	CBA	PFA	CBA	PFA	CBA	PFA	CBA	PFA	CBA	PFA	CBA	PFA	CBA	PFA	CBA	
Reichlin et al. [14]	RCT	105	105	64.0 ± 9.4	63.3 ± 9.6	77/28	74/31	27.0 ± 3.9	27.3 ± 4.7	56	58	13	9	12	16	60.0 ± 7.0	60.0 ± 6.0	2.0 ± 1.5	2.0 ± 1.5	③⑤
Gerstenfeld et al. [15]	RCT	194	83	63.0 ± 8.3	63.2 ± 8.1	121/73	47/36	28.1 ± 4.3	29.7 ± 5.1	89	43	23	14	23	14	60.2 ± 9.2	57.7 ± 10.6	1.8 ± 1.2	1.7 ± 1.3	①②
Rocca et al. [16]	Retrospective	174	348	62.0 ± 11.6	62.1 ± 12.3	110/64	218/130	27.0 ± 4.8	27.2 ± 4.6	78	150	16	24	10	24	59.4 ± 4.2	58.2 ± 7.5	2.0 ± 1.5	2.0 ± 1.5	②③⑥
Chaumont et al. [17]	RCT	151	150	61.0 ± 12.0	63.0 ± 12.0	94/57	93/57	27.1 ± 4.6	26.9 ± 4.2	58	61	13	11	9	6	NR	NR	1.0 ± 1.1	2 ± 1.5	③④
Isenegger et al. [18]	RCT	113	107	65.0 ± 10.4	67.0 ± 8.2	88/25	79/28	27.0 ± 4.4	28.0 ± 4.4	67	70	11	9	12	12	NR	NR	2.0 ± 1.5	3.0 ± 1.5	①②③④⑤⑥
Kueffer et al. [19]	RCT	214	190	69.0 ± 9.6	68.2 ± 11.9	164/50	138/52	28.1 ± 5.5	29.0 ± 5.6	130	129	39	21	44	32	55.0 ± 11.1	55.0 ± 11.1	3.0 ± 1.5	3.0 ± 1.5	③④

*Note:* ① ablation time; ② LA dwell time; ③ atrial arrhythmia recurrence; ④ phrenic paresis; ⑤ release of hypersensitive troponin; ⑥ reconnected PVs.

PFA was performed using the Farapulse PFA system (Boston Scientific Inc. Menlo Park, CA, USA), whereas CBA was performed using either the PolarX Cardiac Cryoablation System (Boston Scientific, Natick, MA, USA) or the Arctic Front Cryoablation System (Medtronic, Minneapolis, MN, USA).

All included studies reported a follow‐up duration of approximately 12 months (range: 12.0 to 12.3 months), with primary efficacy outcomes, including 1‐year arrhythmia‐free survival, assessed at the 12‐month time point. Follow‐up duration was consistent across studies, and key efficacy and safety outcomes were standardized to the 1‐year endpoint. All six studies consistently defined arrhythmia recurrence as atrial arrhythmia lasting at least 30 s after the blanking period. Core monitoring strategies included routine follow‐up visits with 12‐lead electrocardiography (ECG) at predefined time points, typically at 3, 6, and 12 months post‐ablation, supplemented by Holter monitoring in patients with suspected arrhythmia recurrence.

One study, Reichlin et al. [14], used continuous rhythm monitoring with implantable cardiac monitors (ICMs) over 1 year, with daily automated data transmission and scheduled ECG and outpatient visits. Most other studies employed 7‐day Holter monitoring at 3, 6, and 12 months after ablation. Rocca 2024 performed routine clinical assessments and 12‐lead ECG at 1, 3, 6, and 12 months, with 7‐day Holter monitoring reserved for symptom‐driven evaluation.

The six trials demonstrated a low risk of bias in selection, performance, detection, attrition, and reporting domains, with all prespecified outcomes reported according to study protocols (Supporting Information S1: Table 1).

### 3.2 Primary Endpoints

**Figure 2:**
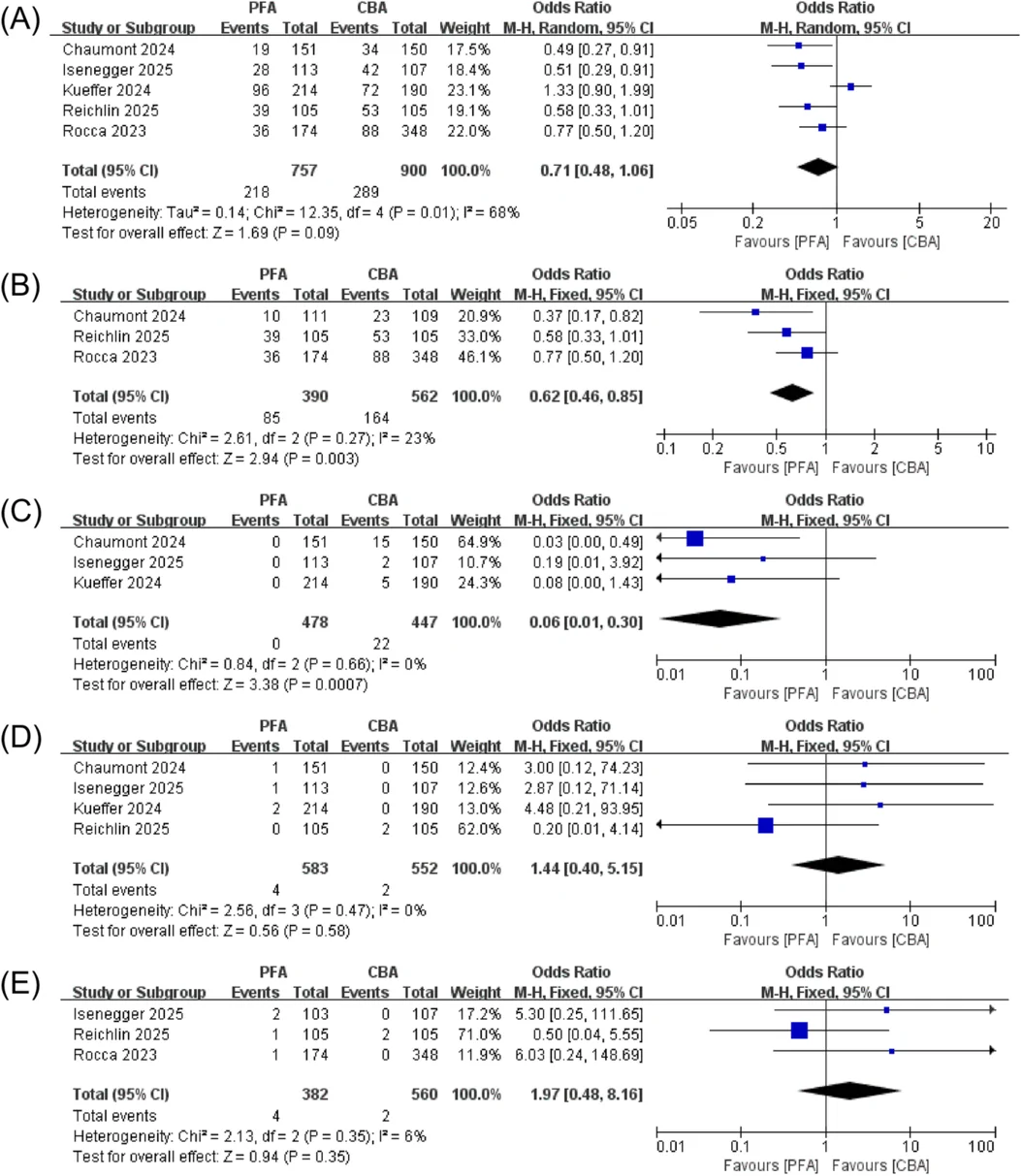
The forest plot displays the ATs recurrence and complications' rates when comparing PFA and CBA for AF. (A) the ATs recurrence in patients with AF, (B) the ATs recurrence in patients with paroxysmal AF, (C) the phrenic paresis, (D) the cardiac tamponade, (E) vascular/cerebrovascular complications. CI, confidence interval; PFA, pulsed field ablation; ATs, atrial tachyarrhythmias; CBA, cryoablation.

No significant difference in AT recurrence was observed between PFA and CBA during follow‐up in patients with AF (OR = 0.71; 95% CI: 0.48 to 1.06; *p* = 0.09; *I* ^2^ = 68%; Figure 2A). However, in patients with paroxysmal AF, the PFA group exhibited a significantly lower incidence of AT recurrence compared with the CBA group (OR = 0.62; 95% CI: 0.46 to 0.85; *p* = 0.003; *I* ^2^ = 23%; Figure 2B).

Additionally, three trials comprising 925 patients reported comparable findings regarding periprocedural complications, demonstrating that CBA was associated with a significantly higher incidence of PNP compared with PFA (OR = 0.06; 95% CI: 0.01 to 0.30; *p* = 0.0007; *I* ^2^ = 0%; Figure 2C). Furthermore, four RCTs reported data on post‐procedural cardiac tamponade, including a total of 1135 patients (583 in the PFA group and 552 in the CBA group). The pooled analysis showed no statistically significant difference in the incidence of cardiac tamponade between the two groups (OR = 1.44; 95% CI: 0.40 to 5.15; *p* = 0.58; Figure 2D), with no heterogeneity across studies (*I* ^2^ = 0%). Three RCTs reported vascular or cerebrovascular complications, including stroke, transient ischemic attack, and cerebrovascular accident, involving a total of 942 patients (382 in the PFA group and 560 in the CBA group). The pooled analysis demonstrated no statistically significant difference in the incidence of these complications between the two groups (OR = 1.97; 95% CI: 0.48 to 8.16; *p* = 0.35; Figure 2E), with low heterogeneity (*I* ^2^ = 6%). Overall, the incidence of these complications was very low in both groups, highlighting the high safety profile of both ablation modalities in clinical practice.

**Figure 3:**
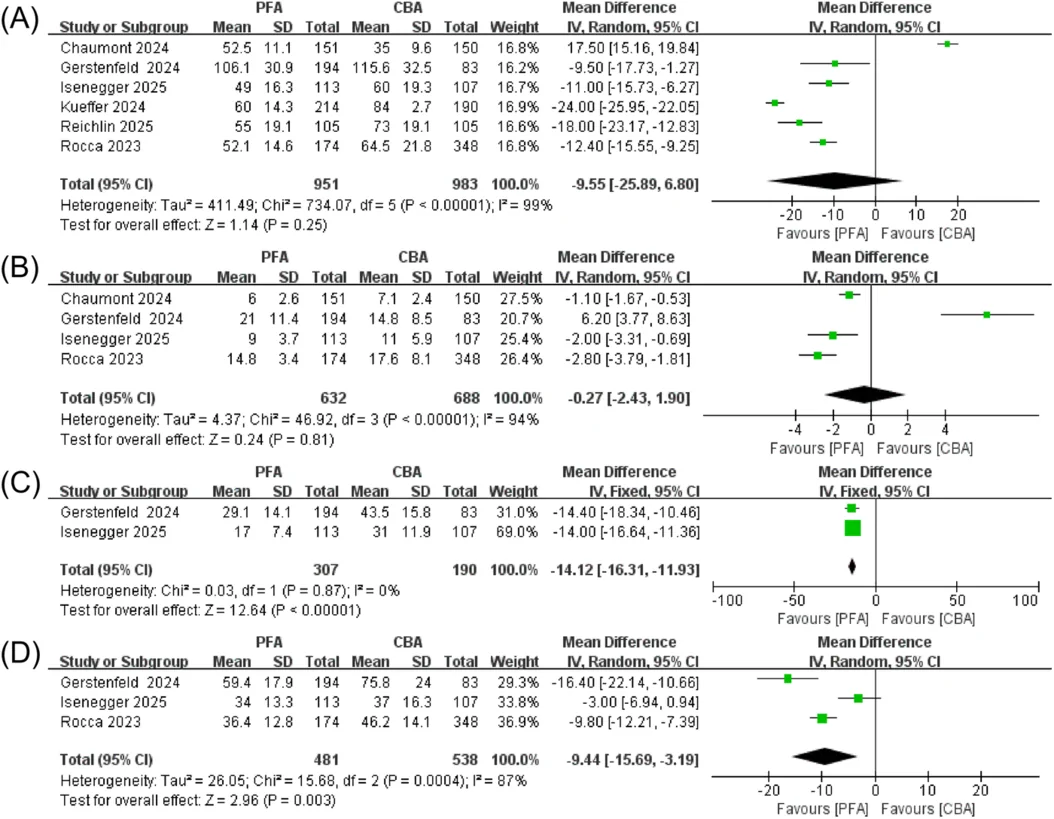
The forest plot displays procedural efficiency metrics when comparing PFA and CBA for AF. (A) the operative time, (B) the fluoroscopy time, (C) the ablation time, (D) the left atria dwell time. CBA, cryoablation; CI, confidence interval; PFA, pulsed field ablation.

All included trials reported procedure time, four reported fluoroscopy time, and ablation time and left atrial dwell time were reported in two and three trials, respectively. No significant difference was observed in procedure time (MD = −9.55; 95% CI: −25.89 to 6.80; *p* = 0.25; *I* ^2^ = 99%; Figure 3A) or fluoroscopy time (MD = −0.27; 95% CI: −2.43 to 1.90; *p* = 0.81; *I* ^2^ = 94%; Figure 3B) between PFA and CBA.

However, PFA was associated with significantly shorter ablation time and left atrial dwell time compared with CBA (MD = −14.12; 95% CI: −16.31 to −11.93; *p* < 0.00001; *I* ^2^ = 0%; Figure 3C) and (MD = −9.44; 95% CI: −15.69 to −3.19; *p* = 0.003; *I* ^2^ = 87%; Figure 3D), respectively.

### 3.3 Secondary Endpoints

**Figure 4:**
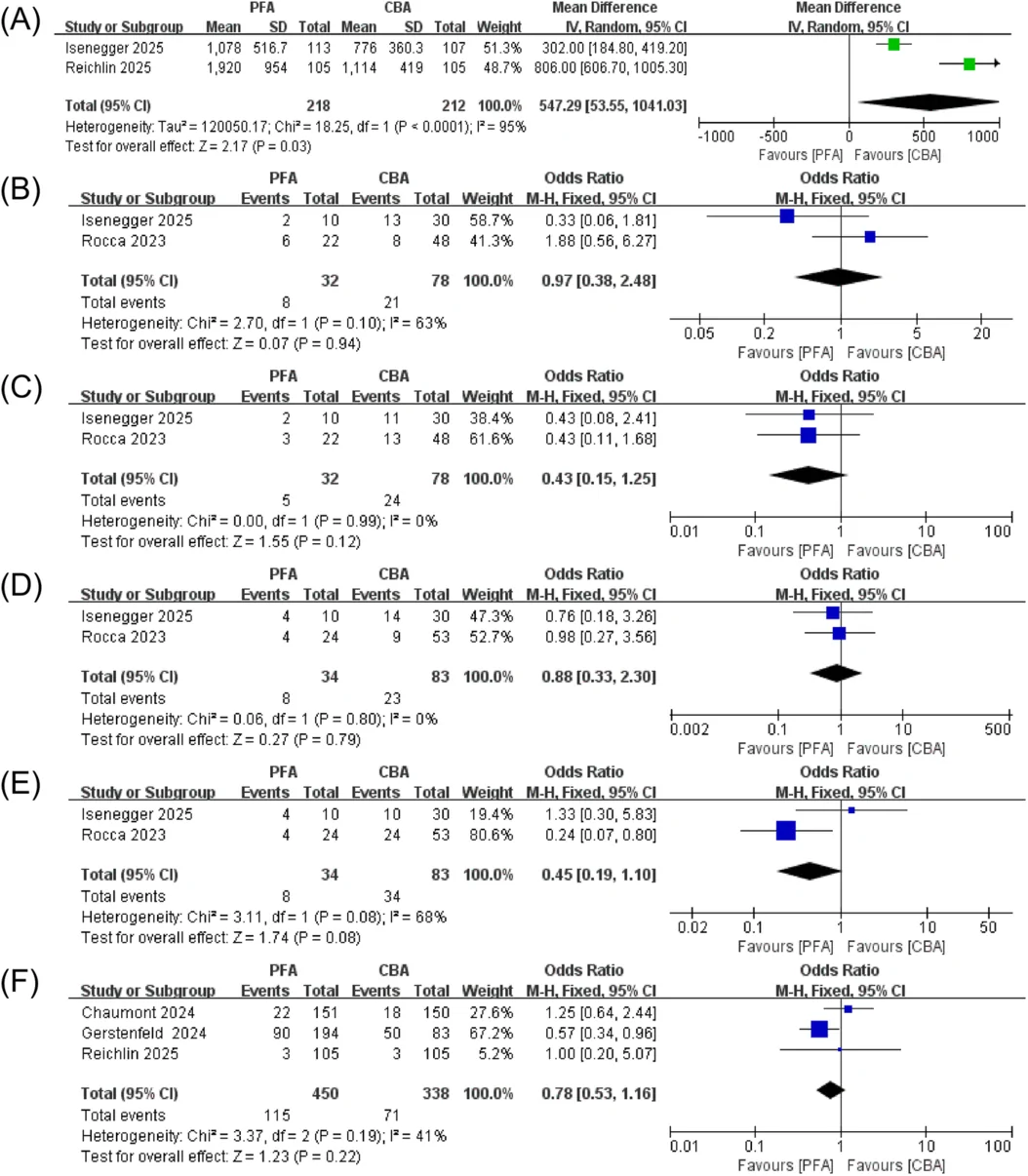
The forest plot displays the secondary endpoints when comparing PFA and CBA for AF. (A) the release of hs‐Tn, (B) left superior PV, (C) left inferior PV, (D) right superior PV, (E) right inferior PV, (F) 12‐month post‐procedural AAD reutilization rates. CBA, cryoablation; CI, confidence interval; hs‐Tn, hypersensitive troponin; PFA, pulsed field ablation; PV, pulmonary vein.

The release of hs‐Tn was significantly higher in the PFA group than in the CBA group (MD = 547.29; 95% CI: 53.55 to 1041.03; *p* = 0.03; *I* ^2^ = 95%; Figure 4A).

PV reconnection is an important indicator for evaluating the effectiveness of ablation techniques. Two trials reported that, after PFA, the left superior PV (LSPV) (8/32; 25%) was the most frequently reconnected vein, followed by the right superior PV (RSPV) (8/34; 23.5%), right inferior PV (RIPV) (8/34; 23.5%), and left inferior PV (LIPV) (5/32; 15.6%).

After CBA, the RIPV was the most frequently reconnected vein, followed by the LIPV (24/78; 30.8%), RSPV (23/83; 27.7%), and LSPV (21/78; 26.9%). No significant differences in reconnection rates were observed between the two groups for LSPV (OR = 0.97; 95% CI: 0.38 to 2.48; *p* = 0.94; *I* ^2^ = 63%; Figure 4B), LIPV (OR = 0.43; 95% CI: 0.15 to 1.25; *p* = 0.12; *I* ^2^ = 0%; Figure 4C), RSPV (OR = 0.88; 95% CI: 0.33 to 2.30; *p* = 0.79; *I* ^2^ = 0%; Figure 4D), and RIPV (OR = 0.45; 95% CI: 0.19 to 1.10; *p* = 0.08; *I* ^2^ = 68%; Figure 4E).

Three RCTs reported data on 12‐month post‐procedural AAD reutilization, including a total of 788 patients (450 in the PFA group and 338 in the CBA group). The pooled analysis demonstrated no statistically significant difference in AAD reutilization rates between the two groups (OR = 0.78; 95% CI: 0.53 to 1.16; Figure 4F), with moderate heterogeneity (*p* = 0.19; *I* ^2^ = 41%). At the individual study level, only the Gerstenfeld 2024 trial reported a significantly lower AAD reutilization rate in the PFA group (OR = 0.57; 95% CI: 0.34 to 0.96), whereas the remaining studies reported no significant differences between groups.

### 3.4 Sensitivity Analysis

**Figure 5:**
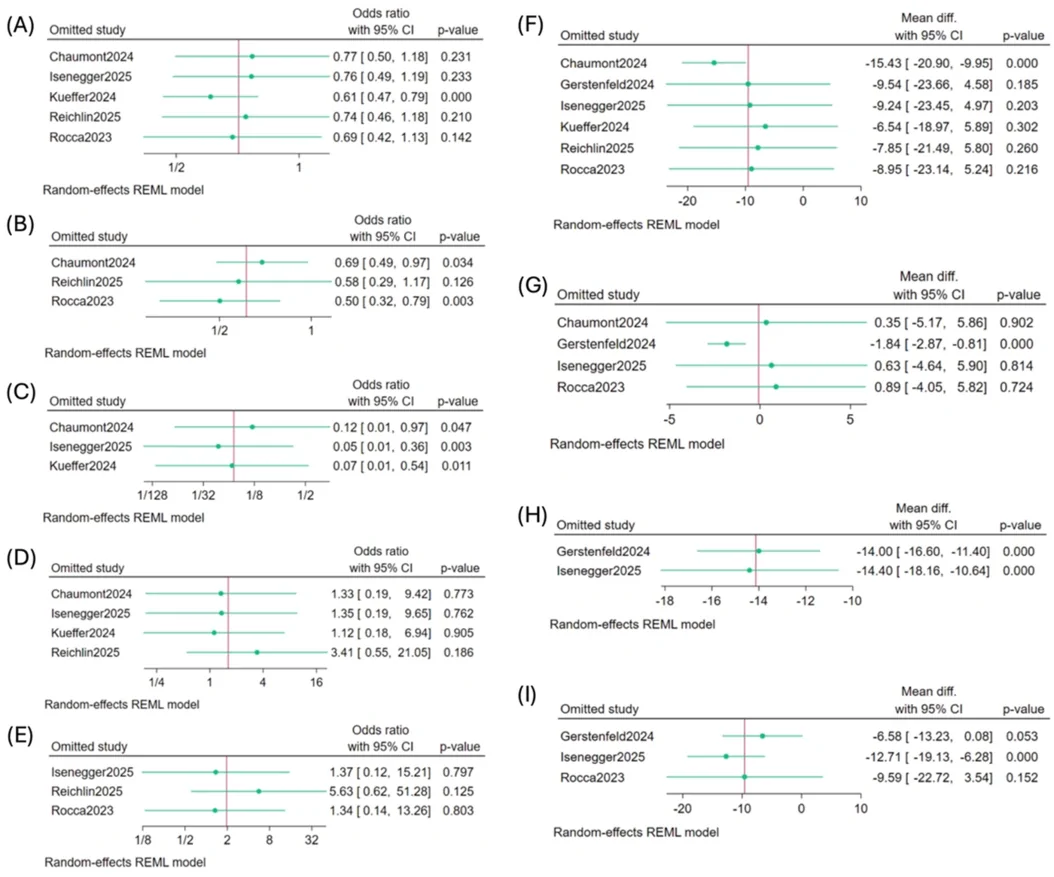
Sensitivity analysis based on the primary endpoints. (A) sensitivity analysis of the ATs recurrence in patients with AF, (B) sensitivity analysis of the ATs recurrence in patients with paroxysmal AF, (C) sensitivity analysis of the phrenic paresis, (D) sensitivity analysis of the cardiac tamponade, (E) sensitivity analysis of vascular/cerebrovascular complications, (F) sensitivity analysis of the operative time, (G) sensitivity analysis of the fluoroscopy time, (H) sensitivity analysis of the ablation time, (I) sensitivity analysis of the left atria dwell time.

Leave‐one‐out sensitivity analyses of the primary endpoints revealed varying degrees of robustness. For the efficacy endpoints, the significance of ATs recurrence in both the AF population and the paroxysmal AF subgroup changed after exclusion of certain individual studies, indicating that these findings were not entirely stable (Figure 5A,B). Analysis of safety‐related outcomes showed that phrenic paresis exhibited good robustness (Figure 5C). By contrast, cardiac tamponade and vascular/cerebrovascular complications remained nonsignificant (Figure 5D,E). Although these results are sensitive to individual studies, the low incidence of events may have limited precision. Among procedural endpoints (Figure 5F–I), operative time and fluoroscopy time were both affected by individual studies and exhibited limited stability. Furthermore, left atrial dwell time was the least stable outcome and was particularly sensitive to study exclusion.

Overall, from the perspective of *p* values, the strongest findings were observed for phrenic paresis and ablation time, whereas results for ATs recurrence, operative time, fluoroscopy time, and especially left atrial dwell time should be interpreted with caution.

### 3.5 Publication Bias

Publication bias was not formally assessed due to the limited number of included studies, as funnel plot asymmetry and Egger's regression test are considered unreliable when fewer than 10 studies are available.

## 4 Discussion

This study presents a meta‐analysis comparing the safety and efficacy of PFA and CBA for PVI in patients with AF. The main findings are as follows:

1. No significant differences were observed in procedure time or fluoroscopy time between PFA and CBA. However, PFA was associated with significantly shorter ablation time and left atrial dwell time.
2. PFA was associated with a lower incidence of AT recurrence in patients with paroxysmal AF.
3. CBA was associated with a significantly higher incidence of PNP compared with PFA.

Based on a comprehensive review of the literature, six recent studies were included in this analysis. The results further support the favorable safety and efficacy profile of PFA compared with CBA. Maintaining catheter stability within a continuously beating heart over prolonged periods remains technically challenging. In addition, tissue edema resulting from prolonged ablation may limit procedural effectiveness [20]. Therefore, the shorter ablation time and reduced left atrial dwell time associated with PFA may not only reduce patient risk but also decrease procedural complexity for electrophysiologists.

In clinical practice, although CBA simplifies ablation procedures and reduces procedural duration, PFA demonstrates additional advantages. For example, Xie et al. [21] demonstrated in animal studies that PFA can effectively ablate myocardial tissue within a shorter time frame. Jonas et al. [22] used a basket or flower‐shaped PFA catheter to perform PVI in 17 patients with paroxysmal AF and 40 patients with persistent AF, achieving a mean ablation time of 65 ± 17 min and a mean fluoroscopy time of 15 ± 5 min.

Similarly, Melanie et al. [23] reported a mean ablation time of 91 ± 30 min for PVI combined with left atrial posterior wall isolation (LAPWI) using the same type of PFA catheter in 59 patients with persistent AF, with a mean fluoroscopy time of 15 ± 7 min. In contrast, Wenzel et al. [24] reported a mean ablation time of 108 ± 25 min for point‐by‐point PVI using a focal ablation catheter in patients with paroxysmal and persistent AF, with a fluoroscopy time of 10 ± 4 min.

Kuck et al. [25] reported mean ablation times of 141 min for RFA and 124 min for CBA. These findings indicate that the mean ablation time associated with PFA is substantially shorter.

Furthermore, the PFA group demonstrated non‐inferiority to the CBA group in terms of the overall recurrence rate of ATs. In addition, PFA may offer particular advantages for AF ablation, especially in patients with paroxysmal AF, compared with CBA.

It is well established that achieving continuous and durable PVI is essential for high‐quality AF ablation, and PVI serves as a key indicator for evaluating AF recurrence following the procedure [26]. RFA achieves PVI through point‐by‐point ablation, whereas the circumferential contact approach of CBA suggests a more homogeneous, reliable, and rapid ablation process. However, despite these theoretical advantages, the frequency of PV reconnection with CBA is comparable to that observed with RFA [27].

Xie et al. [21] demonstrated in animal experiments that the width and depth of lesions created by PFA can be modulated within a certain range by adjusting electrode distance and pulse amplitude, thereby enabling improved control of lesion geometry. Moreover, compared with RFA and CBA, PFA produces more homogeneous and consistent lesions with clearer boundaries and more controlled effects on surrounding tissues.

Each PFA system incorporates catheter designs of different shapes, each providing distinct advantages in terms of ablation efficiency and safety. The six studies included in this meta‐analysis used the Farapulse PFA system, which features a flexible catheter design that facilitates close apposition within the PVs and enhances ablation efficiency. In a series of studies on PFA, Tohoku et al. [28] reported that the rate of PV reconnection was lower when using a 31 mm catheter compared with a 35 mm catheter. Furthermore, a smaller catheter diameter may be associated with a reduced rate of PV reconnection.

In addition, optimization of catheter parameters to modify lesion characteristics or expansion of the ablation area to create more extensive lesions may further reduce recurrence rates. Notably, the release of hs‐Tn following PFA is higher than that observed in the CBA group, suggesting that PFA induces more targeted cardiomyocyte injury.

CA induces cardiomyocyte injury and triggers an inflammatory response. Increasing evidence indicates that inflammation is closely associated with the development, persistence, and recurrence of AF [29, 30]. Liu et al. [31] demonstrated that PFA induces a milder inflammatory response compared with CBA.

The differing mechanisms of action of CBA and PFA may explain this observation. CBA utilizes the thermodynamic properties of argon gas, which absorbs heat during rapid expansion, generating cryogenic temperatures that result in cellular injury and death. This cryoinjury process typically occurs in three stages [32]. First, freezing induces irreversible cellular damage. Second, thawing leads to ischemic necrosis. Finally, the affected tissue is replaced by fibrotic tissue.

In contrast, PFA induces IRE of cardiomyocytes, which contributes to its favorable safety profile. IRE involves the application of short bursts of high‐frequency pulsed current that disrupt the cell membrane of cardiomyocytes, resulting in structural damage or denaturation of membrane proteins [13]. The lipid bilayer is unable to reseal after removal of the electric field [33, 34].

Previous studies have demonstrated that different tissues exhibit distinct thresholds for transmembrane voltage during electroporation [35, 36]. When cells are exposed to electric fields below the critical threshold, membrane permeability is temporary and reversible. However, when the electric field exceeds this threshold, permeabilization becomes irreversible, leading to apoptosis [37].

Bradley et al. [38] reported that cardiomyocytes have a lower threshold for pulse intensity compared with other tissue types, with a minimum effective intensity of approximately 375 to 400 V/cm. This characteristic allows electrophysiologists to optimize PFA parameters to achieve effective ablation while minimizing injury to adjacent tissues.

In summary, CBA relies on extreme temperatures to induce tissue injury and necrosis, resulting in a more pronounced and widespread inflammatory response. In contrast, PFA primarily induces apoptosis, preserves the extracellular matrix, and reduces structural remodeling [39]. The tissue selectivity of PFA further limits inflammation in surrounding tissues, thereby minimizing collateral damage and improving patient outcomes.

The comprehensive analysis of periprocedural and post‐procedural complications clarifies the distinct safety profiles of PFA and CBA for AF, with consistent findings supporting the clinical value of PFA. A key observation is the significant advantage of PFA in reducing PNP. This difference arises from the fundamental mechanisms of the two ablation modalities. CBA exerts its effects through cryogenic thermal injury, which carries a risk of phrenic nerve damage during PVI. In contrast, PFA utilizes non‐thermal electroporation, a technique characterized by high tissue selectivity that enables precise ablation of cardiomyocytes while sparing adjacent neural tissue, thereby minimizing the risk of PNP.

From a clinical perspective, the safety profile of PFA offers meaningful benefits. PNP, even when transient, may lead to dyspnea and prolonged hospitalization. Therefore, the ability of PFA to reduce this complication supports its preferential use in patients with anatomical features associated with increased PNP risk. Meanwhile, comparable rates of severe complications indicate that PFA does not compromise overall safety while reducing PNP, consistent with previous trials and meta‐analyses. However, the relatively small number of trials and the low incidence of severe complications limit the statistical power to detect subtle differences.

In summary, PFA maintains similarly low rates of severe non‐specific complications compared with CBA while significantly reducing procedure‐specific PNP, supporting its role as a safe and effective alternative for AF ablation, particularly in patients at high risk of PNP.

The favorable safety profile of PFA supports its broader application in ablation procedures. Reddy et al. and Melanie et al. [23, 40] investigated PVI combined with LAPWI in patients with persistent AF and demonstrated the feasibility and safety of PFA‐guided LAPWI. However, the use of prophylactic LAPWI in combination with PVI remains controversial. Left atrial electrical isolation is considered a promising therapeutic strategy for AF.

Reddy et al. [41] evaluated a novel device that integrates mechanical left atrial appendage occlusion (LAAO) with left atrial electrical isolation using PFA (LAAEI). At 6‐month follow‐up, patients exhibited no recurrence of AF and no adverse events requiring intervention.

A previous meta‐analysis by Vetta et al. [10] evaluated 20 studies on PFA and 70 studies on CBA, including a total of 16 247 patients undergoing AF ablation, of whom 2481 received PFA and 13 766 underwent CBA. Their analysis assessed procedural success, periprocedural complications, procedure duration, fluoroscopy time, and freedom from ATs at 1 year. Although their findings indicated comparable safety between the two modalities, several differences exist between their study and the present analysis.

First, no significant differences in procedure time or fluoroscopy time were observed in our study, whereas PFA demonstrated advantages in ablation time and left atrial dwell time. Second, the evaluation indices differed. The present study included hs‐Tn as a perioperative safety marker and focused on PVI as a key indicator of ablation efficacy at 1‐year follow‐up. Third, this analysis prioritized recently published RCTs up to March 2025, incorporating five additional studies to reflect the most current evidence.

Furthermore, this study complements and refines the meta‐analysis by Zhang et al. [42] through several methodological differences. Only PFA procedures using pentaspline catheters (FARAWAVE) were included to enhance intervention homogeneity, whereas Zhang et al. included all PFA catheter types. With an extended search period to March 2025, five additional RCTs were incorporated, and subgroup analysis in patients with paroxysmal AF identified a significant reduction in AT recurrence (OR = 0.62; 95% CI: 0.46 to 0.85; *p* = 0.003), which was not examined in the previous study.

Additional endpoints, including left atrial dwell time and 12‐month AAD reutilization, were also analyzed to better inform clinical decision‐making. Both studies demonstrated the superior safety of PFA in reducing PNP. However, Zhang et al. reported a higher risk of cardiac tamponade with PFA, likely due to the inclusion of early observational studies with heterogeneous protocols, whereas the present RCT‐focused analysis showed no such difference (OR = 1.44; 95% CI: 0.40 to 5.15; *p* = 0.58).

Overall, the catheter‐specific and subgroup‐focused design of this study complements previous broader analyses and provides more precise clinical insights, particularly for patients with paroxysmal AF.

Therefore, when selecting an ablation energy modality, PFA may represent a preferable option due to its favorable safety profile. Further studies are warranted to evaluate its performance in the treatment of other arrhythmias.

## 5 Limitations

This study has several limitations.

First, only six studies were included in this meta‐analysis, resulting in a relatively small sample size. Multicenter, large‐scale prospective studies are required. As PFA is an emerging technique for AF ablation, comparative studies between PFA and CBA are ongoing, and additional data are expected to become available.

Second, the included studies involved heterogeneous patient populations and varied study designs, which may limit the generalizability of the findings.

Third, substantial heterogeneity existed in monitoring strategies, including the use of ECG, Holter monitoring, event monitoring, and device interrogation at different time intervals, which may have influenced the assessment of arrhythmia recurrence.

Fourth, all included studies exclusively used the FARAPULSE system, limiting the applicability of these findings to other PFA systems. Different systems employ distinct waveforms and energy settings, which may result in different clinical outcomes.

Fifth, sensitivity analyses revealed variations in the robustness of the primary endpoints. The instability observed in ATs recurrence may partly reflect differences in baseline patient characteristics and follow‐up strategies across the included studies. In addition, procedure‐related endpoints are more susceptible to between‐study variability, including factors such as operator experience and procedural techniques. Therefore, although the results for phrenic paresis and ablation time are relatively stable, the findings for other primary endpoints should be interpreted with caution.

Sixth, the potential for publication bias could not be reliably evaluated due to the small number of included studies, which may affect the robustness of the findings.

Further large‐scale RCTs are necessary to validate these findings.

## 6 Conclusion

The findings of this meta‐analysis indicate that PFA is associated with shorter ablation time and left atrial dwell time compared with CBA, while demonstrating non‐inferiority in procedure time and fluoroscopy time. Although no significant difference was observed in the overall recurrence rates of ATs, patients treated with PFA exhibited lower recurrence rates, particularly among those with paroxysmal AF, suggesting its potential to achieve improved rhythm outcomes.

From a safety perspective, PFA demonstrated greater tissue selectivity, as reflected by the absence of PV stenosis. Given these advantages, PFA is expected to be increasingly applied in the treatment of arrhythmias beyond AF and may become a standard approach for CA in the future.

## Ethics Statement

The authors have nothing to report.

## Conflicts of Interest

The authors declare no conflicts of interest.

## Supporting information


Supporting File

## Data Availability

The data that support the findings of this study are available from the corresponding author upon reasonable request. All data generated or analyzed during this study are included in this article and its supporting informations.

## References

[1] J. A. Joglar , M. K. Chung , A. L. Armbruster , et al., “2023 ACC/AHA/ACCP/HRS Guideline for the Diagnosis and Management of Atrial Fibrillation: A Report of the American College of Cardiology/American Heart Association Joint Committee on Clinical Practice Guidelines,” Circulation 149, no. 1 (January 2024): e1–e156. 38033089 10.1161/CIR.0000000000001193 PMC11095842

[2] G. Lippi , F. Sanchis‐Gomar , and G. Cervellin , “Global Epidemiology of Atrial Fibrillation: An Increasing Epidemic and Public Health Challenge,” International Journal of Stroke 16 (2021): 217–221. 31955707 10.1177/1747493019897870

[3] H. F. Tse , Y. J. Wang , M. Ahmed Ai‐Abdullah , et al., “Stroke Prevention in Atrial Fibrillation‐‐An Asian Stroke Perspective,” Heart Rhythm: The Official Journal of the Heart Rhythm Society 10, no. 7 (July 2013): 1082–1088. 10.1016/j.hrthm.2013.03.017 23501173

[4] B. J. J. M. Brundel , X. Ai , M. T. Hills , M. F. Kuipers , G. Y. H. Lip , and N. M. S. de Groot , “Atrial Fibrillation,” Nature Reviews Disease Primers 8, no. 1 (April 2022): 21. 10.1038/s41572-022-00347-9 35393446

[5] C. Blomström‐Lundqvist , S. Gizurarson , J. Schwieler , et al., “Effect of Catheter Ablation vs Antiarrhythmic Medication on Quality of Life in Patients With Atrial Fibrillation: The CAPTAF Randomized Clinical Trial,” Journal of the American Medical Association 321, no. 11 (March 2019): 1059–1068. 30874754 10.1001/jama.2019.0335 PMC6439911

[6] F. D. Ramirez , V. Y. Reddy , R. Viswanathan , M. Hocini , and P. Jaïs , “Emerging Technologies for Pulmonary Vein Isolation,” Circulation Research 127, no. 1 (2020): 170–183. 32716722 10.1161/CIRCRESAHA.120.316402

[7] T. J. Buist , D. P. Zipes , and A. Elvan , “Atrial Fibrillation Ablation Strategies and Technologies: Past, Present, and Future,” Clinical Research in Cardiology 110, no. 6 (2021): 775–788. 33089361 10.1007/s00392-020-01751-5

[8] R. Latchamsetty and F. Morady , “Atrial Fibrillation Ablation,” Annual Review of Medicine 69 (2018): 53–63. 10.1146/annurev-med-041316-090015 28806148

[9] A. Verma , S. J. Asivatham , T. Deneke , Q. Castellvi , and R. E. Neal , “Primer on Pulsed Electrical Field Ablation: Understanding the Benefits and Limitations,” Circulation: Arrhythmia and Electrophysiology 14, no. 9 (September 2021): e010086. 34538095 10.1161/CIRCEP.121.010086

[10] A. Verma , D. E. Haines , L. V. Boersma , et al., “Pulsed Field Ablation for the Treatment of Atrial Fibrillation: PULSED AF Pivotal Trial,” Circulation 147, no. 19 (2023): 1422–1432. 36877118 10.1161/CIRCULATIONAHA.123.063988 PMC10158608

[11] O. M. Aldaas , C. Malladi , F. T. Han , et al., “Pulsed Field Ablation Versus Thermal Energy Ablation for Atrial Fibrillation: A Systematic Review and Meta‐Analysis of Procedural Efficiency, Safety, and Efficacy,” Journal of Interventional Cardiac Electrophysiology 67, no. 3 (2024): 639–648. 37855992 10.1007/s10840-023-01660-3 PMC11016003

[12] J. C. Chen , Y. J. Mao , Q. Y. Huang , et al., “Meta‐Analysis of Thermal Versus Pulse Field Ablation for Pulmonary Vein Isolation Durability in Atrial Fibrillation: Insights From Repeat Ablation,” Clinical Cardiology 48, no. 6 (2025): e70151. 40437855 10.1002/clc.70151 PMC12120194

[13] G. Vetta , D. G. Della Rocca , A. Parlavecchio , et al., “Multielectrode Catheter‐Based Pulsed Electric Field Vs. Cryoballoon for Atrial Fibrillation Ablation: A Systematic Review and Meta‐Analysis,” Europace: European Pacing, Arrhythmias, and Cardiac Electrophysiology: Journal of the Working Groups on Cardiac Pacing, Arrhythmias, and Cardiac Cellular Electrophysiology of the European Society of Cardiology 26, no. 12 (2024): euae293. 39579376 10.1093/europace/euae293 PMC11641428

[14] T. Reichlin , T. Kueffer , P. Badertscher , et al., “Pulsed Field or Cryoballoon Ablation for Paroxysmal Atrial Fibrillation,” New England Journal of Medicine 392, no. 15 (2025): 1497–1507. 40162734 10.1056/NEJMoa2502280

[15] E. P. Gerstenfeld , M. Mansour , W. Whang , et al., “Autonomic Effects of Pulsed Field vs Thermal Ablation for Treating Atrial Fibrillation,” JACC: Clinical Electrophysiology 10, no. 7 Pt 2 (2024): 1634–1644. 38869507 10.1016/j.jacep.2024.05.005

[16] D. G. Della Rocca , L. Marcon , M. Magnocavallo , et al., “Pulsed Electric Field, Cryoballoon, and Radiofrequency for Paroxysmal Atrial Fibrillation Ablation: A Propensity Score‐Matched Comparison,” Europace: European Pacing, Arrhythmias, and Cardiac Electrophysiology: Journal of the Working Groups on Cardiac Pacing, Arrhythmias, and Cardiac Cellular Electrophysiology of the European Society of Cardiology 26, no. 1 (2023): euae016. 38245007 10.1093/europace/euae016 PMC10823352

[17] C. Chaumont , C. Hayoun , A. Savoure , et al., “Pentaspline Pulsed Field Ablation Catheter Versus Cryoballoon for Atrial Fibrillation Ablation: Results From a Prospective Comparative Study,” Journal of the American Heart Association 13, no. 6 (2024): e033146. 38471838 10.1161/JAHA.123.033146 PMC11010030

[18] C. Isenegger , R. Arnet , F. Jordan , et al., “Pulsed‐Field Ablation Versus Cryoballoon Ablation in Patients With Persistent Atrial Fibrillation,” IJC Heart & Vasculature 59 (2025): 101684. 40371321 10.1016/j.ijcha.2025.101684 PMC12076779

[19] T. Kueffer , R. Stettler , J. Maurhofer , et al., “Pulsed‐Field Vs Cryoballoon vs Radiofrequency Ablation: Outcomes After Pulmonary Vein Isolation in Patients With Persistent Atrial Fibrillation,” Heart Rhythm: the Official Journal of the Heart Rhythm Society 21, no. 8 (2024): 1227–1235. 10.1016/j.hrthm.2024.04.045 38614191

[20] E. Leshem , I. Zilberman , C. M. Tschabrunn , et al., “High‐Power and Short‐Duration Ablation for Pulmonary Vein Isolation,” JACC: Clinical Electrophysiology 4, no. 4 (2018): 467–479. 30067486 10.1016/j.jacep.2017.11.018

[21] F. Xie , F. Varghese , A. G. Pakhomov , et al., “Ablation of Myocardial Tissue With Nanosecond Pulsed Electric Fields,” PLoS One 10, no. 12 (December 2015): e0144833. 26658139 10.1371/journal.pone.0144833 PMC4687652

[22] J. Wörmann , J. H. Schipper , J. Lüker , et al., “Comparison of Pulsed‐Field Ablation Versus Very High Power Short Duration‐Ablation for Pulmonary Vein Isolation,” Journal of Cardiovascular Electrophysiology 34, no. 12 (2023): 2417–2424. 37846194 10.1111/jce.16101

[23] M. A. Gunawardene , G. Frommeyer , C. Ellermann , et al., “Left Atrial Posterior Wall Isolation With Pulsed Field Ablation in Persistent Atrial Fibrillation,” Journal of Clinical Medicine 12, no. 19 (September 2023): 6304. 37834948 10.3390/jcm12196304 PMC10573684

[24] J. P. Wenzel , M. D. Lemoine , L. Rottner , et al., “Nonthermal Point‐by‐Point Pulmonary Vein Isolation Using a Novel Pulsed Field Ablation System,” Circulation: Arrhythmia and Electrophysiology 16, no. 9 (Septembe 2023): e012093. 37638409 10.1161/CIRCEP.123.012093

[25] K. H. Kuck , J. Brugada , A. Fürnkranz , et al., “Cryoballoon or Radiofrequency Ablation for Paroxysmal Atrial Fibrillation,” New England Journal of Medicine 374, no. 23 (2016): 2235–2245. 27042964 10.1056/NEJMoa1602014

[26] F. Ouyang , M. Antz , S. Ernst , et al., “Recovered Pulmonary Vein Conduction as a Dominant Factor for Recurrent Atrial Tachyarrhythmias After Complete Circular Isolation of the Pulmonary Veins: Lessons From Double Lasso Technique,” Circulation 111 (2005): 127–135. 15623542 10.1161/01.CIR.0000151289.73085.36

[27] F. D. Ramirez , V. Y. Reddy , R. Viswanathan , M. Hocini , and P. Jaïs , “Emerging Technologies for Pulmonary Vein Isolation,” Circulation Research 127, no. 1 (2020): 170–183. 32716722 10.1161/CIRCRESAHA.120.316402

[28] S. Tohoku , K. R. J. Chun , S. Bordignon , et al., “Findings From Repeat Ablation Using High‐Density Mapping After Pulmonary Vein Isolation With Pulsed Field Ablation,” EP Europace 25, no. 2 (February 2023): 433–440. 10.1093/europace/euac211 PMC9935020 36427201

[29] M. Sagris , E. P. Vardas , P. Theofilis , A. S. Antonopoulos , E. Oikonomou , and D. Tousoulis , “Atrial Fibrillation: Pathogenesis, Predisposing Factors, and Genetics,” International Journal of Molecular Sciences 23, no. 1 (December 2021): 6. 35008432 10.3390/ijms23010006 PMC8744894

[30] E. Ozkan , D. Elcik , S. Barutcu , et al., “Inflammatory Markers as Predictors of Atrial Fibrillation Recurrence: Exploring the C‐Reactive Protein to Albumin Ratio in Cryoablation Patients,” Journal of Clinical Medicine 12, no. 19 (2023 Sep 30): 6313. 37834958 10.3390/jcm12196313 PMC10573371

[31] D. Liu , Y. Li , and Q. Zhao , “Effects of Inflammatory Cell Death Caused by Catheter Ablation on Atrial Fibrillation,” Journal of Inflammation Research 16 (August 2023): 3491–3508. 37608882 10.2147/JIR.S422002 PMC10441646

[32] P. Khairy and M. Dubuc , “Transcatheter Cryoablation Part I: Preclinical Experience,” Pacing and Clinical Electrophysiology 31, no. 1 (2008): 112–120. 18181919 10.1111/j.1540-8159.2007.00934.x

[33] W. Chen , “Electroconformational Denaturation of Membrane Proteins,” Annals of the New York Academy of Sciences 1066 (2006): 92–105. 10.1196/annals.1363.028 16533921

[34] L. Rubinsky , E. Guenther , P. Mikus , M. Stehling , and B. Rubinsky , “Electrolytic Effects During Tissue Ablation by Electroporation,” Technology in Cancer Research & Treatment 15, no. 5 (October 2016): NP95–NP103. 10.1177/1533034615601549 26323571

[35] F. D. Ramirez , V. Y. Reddy , R. Viswanathan , M. Hocini , and P. Jaïs , “Emerging Technologies for Pulmonary Vein Isolation,” Circulation Research 127, no. 1 (2020 Jun 19): 170–183. 32716722 10.1161/CIRCRESAHA.120.316402

[36] A. Al‐Khadra , V. Nikolski , and I. R. Efimov , “The Role of Electroporation in Defibrillation,” Circulation Research 87, no. 9 (2000): 797–804. 11055984 10.1161/01.res.87.9.797

[37] I. Kaminska , M. Kotulska , A. Stecka , et al., “Electroporation‐Induced Changes in Normal Immature Rat Myoblasts (H9C2),” General Physiology and Biophysics 31, no. 1 (2012): 19–25. 22447827 10.4149/gpb_2012_003

[38] C. J. Bradley and D. E. Haines , “Pulsed Field Ablation for Pulmonary Vein Isolation in the Treatment of Atrial Fibrillation,” Journal of Cardiovascular Electrophysiology 31, no. 8 (August 2020): 2136–2147. 32107812 10.1111/jce.14414

[39] T. Batista Napotnik , T. Polajžer , and D. Miklavčič , “Cell Death Due to Electroporation—A Review,” Bioelectrochemistry 141 (2021): 107871. 34147013 10.1016/j.bioelechem.2021.107871

[40] V. Y. Reddy , A. Anic , J. Koruth , et al., “Pulsed Field Ablation in Patients With Persistent Atrial Fibrillation,” Journal of the American College of Cardiology 76 (2020): 1068–1080. 32854842 10.1016/j.jacc.2020.07.007

[41] M. Tang , X. Wang , and V. Y. Reddy , “Simultaneous Pulsed Field Ablation and Mechanical Closure of Left Atrial Appendage Using a Novel Device,” JACC: Case Reports 22 (2023): 101963. 37790777 10.1016/j.jaccas.2023.101963 PMC10544081

[42] H. Zhang , H. Zhang , H. Lu , Y. Mao , and J. Chen , “Meta‐Analysis of Pulsed‐Field Ablation Versus Cryoablation for Atrial Fibrillation,” Pacing and Clinical Electrophysiology 47, no. 5 (2024): 603–613. 38525525 10.1111/pace.14971

