# Supplementary material for: Safety and Efficacy of Pulsed‐Field Ablation With a Pentaspline Ablation Catheter Versus Cryoablation for Atrial Fibrillation: A Systematic Review and Meta‐Analysis
Source: Clin Cardiol. 2026 Aug 30;49(9):e70459. doi: 10.1002/clc.70459 (PMC13527216; doi:10.1002/clc.70459)
Supplement: Supplementary file 1 [file CLC-49-e70459-s001.docx]

**Table S1** Newcastle-Ottawa Scale assessment results of the six studies

| **Author name: Reichlin (2025)** | |
| --- | --- |
| **Items** | **Response options** |
| Selection | |
| *Representativeness of the exposed cohort* | *** |
| *Selection of the non exposed cohort* | *** |
| *Ascertainment of exposure* | *** |
| *Demonstration that outcome of interest was not present at start of study* | *** |
| Comparability | |
| *Comparability of cohorts on the basis of the design or analysis* | *** |
| Outcome | |
| *Assessment of outcome* | *** |
| *Was follow-up long enough for outcomes to occur* | *** |
| *Adequacy of follow up of cohorts* | *** |

| **Author name: Gerstenfeld (2024)** | |
| --- | --- |
| **Items** | **Response options** |
| Selection | |
| *Representativeness of the exposed cohort* | *** |
| *Selection of the non exposed cohort* | *** |
| *Ascertainment of exposure* | *** |
| *Demonstration that outcome of interest was not present at start of study* | *** |
| Comparability | |
| *Comparability of cohorts on the basis of the design or analysis* | **** |
| Outcome | |
| *Assessment of outcome* | *** |
| *Was follow-up long enough for outcomes to occur* | *** |
| *Adequacy of follow up of cohorts* | *** |

| **Author name: Rocca (2023)** | |
| --- | --- |
| **Items** | **Response options** |
| Selection | |
| *Representativeness of the exposed cohort* | *** |
| *Selection of the non exposed cohort* | *** |
| *Ascertainment of exposure* | *** |
| *Demonstration that outcome of interest was not present at start of study* | *** |
| Comparability | |
| *Comparability of cohorts on the basis of the design or analysis* | **** |
| Outcome | |
| *Assessment of outcome* | *** |
| *Was follow-up long enough for outcomes to occur* |  |
| *Adequacy of follow up of cohorts* | *** |

| **Author name: Chaumont (2024)** | |
| --- | --- |
| **Items** | **Response options** |
| Selection | |
| *Representativeness of the exposed cohort* | *** |
| *Selection of the non exposed cohort* | *** |
| *Ascertainment of exposure* | *** |
| *Demonstration that outcome of interest was not present at start of study* | *** |
| Comparability | |
| *Comparability of cohorts on the basis of the design or analysis* | **** |
| Outcome | |
| *Assessment of outcome* | *** |
| *Was follow-up long enough for outcomes to occur* | *** |
| *Adequacy of follow up of cohorts* | *** |

| **Author name: Isenegger (2025)** | |
| --- | --- |
| **Items** | **Response options** |
| Selection | |
| *Representativeness of the exposed cohort* | *** |
| *Selection of the non exposed cohort* | *** |
| *Ascertainment of exposure* | *** |
| *Demonstration that outcome of interest was not present at start of study* | *** |
| Comparability | |
| *Comparability of cohorts on the basis of the design or analysis* | **** |
| Outcome | |
| *Assessment of outcome* | *** |
| *Was follow-up long enough for outcomes to occur* | *** |
| *Adequacy of follow up of cohorts* |  |

| **Author name: Kueffer (2024)** | |
| --- | --- |
| **Items** | **Response options** |
| Selection | |
| *Representativeness of the exposed cohort* | *** |
| *Selection of the non exposed cohort* | *** |
| *Ascertainment of exposure* | *** |
| *Demonstration that outcome of interest was not present at start of study* | *** |
| Comparability | |
| *Comparability of cohorts on the basis of the design or analysis* | *** |
| Outcome | |
| *Assessment of outcome* | *** |
| *Was follow-up long enough for outcomes to occur* | * |
| *Adequacy of follow up of cohorts* | *** |

Studies with scores of 0-3, 4-6, 7-9 were, respectively, considered as low, moderate, and high quality.
